# Impulsivity and reward sensitivity facets as predictors of weight change in children: Differences by binge‐eating disorder diagnostic status

**DOI:** 10.1111/ijpo.12987

**Published:** 2022-10-19

**Authors:** Alondra Valdez, Kathryn E. Smith, Tyler B. Mason

**Affiliations:** ^1^ Department of Family and Consumer Sciences California State University Northridge California USA; ^2^ Department of Psychiatry and Behavioral Sciences University of Southern California Los Angeles California USA; ^3^ Department of Population and Public Health Sciences University of Southern California Los Angeles California USA

**Keywords:** binge‐eating disorder, children, impulsivity, obesity, reward sensitivity

## Abstract

**Background:**

Impulsivity and reward sensitivity are personality traits associated with obesity and binge‐eating disorder (BED), but little research has examined prospective associations between these traits and body mass index *z*‐score (BMI‐*z*) differentially for children with and without BED.

**Objective:**

Using data from the Adolescent Brain and Cognitive Development study, the present analysis examined predictive associations between impulsivity and reward sensitivity facets with BMI‐*z* 1 year later in children with versus without BED.

**Methods:**

Nine‐to‐ten‐year‐old children were recruited across the United States and completed self‐report questionnaires and clinical interviews at baseline, and height and weight were taken at baseline and 1‐year follow‐up. Multivariable models were run with baseline impulsivity and reward sensitivity facets predicting 1‐year BMI‐*z* in children with versus without BED.

**Results:**

Reward sensitivity and impulsivity were unrelated to BMI‐*z* at follow‐up in children without BED. Greater negative urgency (i.e., proclivity to act rashly without forethought in response to negative emotions) and lower positive urgency (i.e., proclivity to act rashly without forethought due to positive emotions) predicted increased BMI‐*z* at 1‐year follow‐up in children with BED subsample.

**Conclusions:**

Findings indicate the importance of developing theoretical models and interventions for obesity prevention in children with BED that address emotional impulsivity.

## 1 INTRODUCTION

Childhood obesity has increased globally in the past few decades from 4% in 1976 to 18% in 2016.^1^ Obesity prevention is particularly important in childhood because of its health implications across the lifespan, including increased risk for heart disease, early death, and disability.^1^, ^2^, ^3^ As such, due to the continuing high rates of obesity in children, research informing the development of novel interventions to prevent childhood obesity is needed. Studying personality‐based mechanisms underlying children's weight gain may be useful to inform extant theoretical models and development of tailored preventions. Specifically, two broad‐based personality traits associated with obesity risk include impulsivity and reward sensitivity.^4^, ^5^, ^6^ Impulsivity and reward sensitivity are particularly relevant to study in childhood given that they have been shown to be developmentally sensitive, especially during adolescence.^7^, ^8^

### 1.1 IMPULSIVITY, REWARD SENSITIVITY, AND OBESITY IN CHILDREN

#### 1.1.1 Impulsivity and obesity

Impulsivity is a multidimensional construct that involves decision‐making without forethought and impairments in self‐control.^9^, ^10^ As demonstrated by a meta‐analytic review that included data from over 3000 participants aged 2–21, impulsivity is positively correlated with higher BMI.^5^ Although impulsivity has been operationalized in many ways, a widely used multidimensional model of impulsivity is based on the UPPS‐P Impulsive Behaviour Scale. The UPPS‐P includes five subscales: positive urgency (i.e., the proclivity for rash action when feeling positive emotion), negative urgency (i.e., the proclivity for rash action when feeling negative emotion), lack of premeditation (i.e., the proclivity to act without thinking of the consequences first), lack of perseverance (i.e., the proclivity to have difficulty staying focused on a task), and sensation seeking (i.e., the proclivity to pursue new and thrilling experiences).^11^, ^12^ Using this scale in a cross‐sectional study of adolescents, Smith et al.^13^ found that these subscales were differentially associated with energy‐balance behaviours, including unhealthy dietary intake and infrequent vigorous exercise. Specifically, higher sensation seeking, positive urgency, and negative urgency were associated with unhealthy dietary intake, while higher negative urgency and lack of perseverance were associated with less vigorous exercise. Further, higher sensation seeking, positive urgency, and lack of premeditation were found to be closely linked to more frequent vigorous exercise. Thus, these five subscales may have implications for energy‐balance behaviours and ultimately weight gain.

#### 1.1.2 Reward sensitivity and obesity

Reward sensitivity includes one's unique disposition towards engaging in behaviours to reach a positive stimulus.^14^ There have been mixed results regarding associations between reward sensitivity and BMI in children, with some studies finding a positive association and others finding no relationship.^15^, ^16^, ^17^, ^18^ A key reward sensitivity measure developed by Carver and White^19^ includes two systems of behavioural motivation. The first (i.e., the behavioural inhibition system, BIS) is concerned with regulating feelings of disinterest. The other system is the behavioural approach system (BAS), which is concerned with regulating feelings of desire towards completing a goal. The BAS includes two key components: reward responsiveness (i.e., the propensity to have a positive response surrounding rewarding experiences) and drive (i.e., the propensity to pursue a goal tenaciously).

Subscales from the BIS/BAS scale have been shown to be associated with various energy‐balance behaviours. De Cock et al.^20^ found that the drive and reward responsiveness subscales were positively correlated with unhealthy snacking in 14‐ to 16‐year‐olds, and reward responsiveness was also positively correlated with healthy snacking. De Decker et al.^15^ also found a positive correlation between drive and unhealthy food consumption in 5.5‐ to 12‐year‐olds. Using a related measure of reward sensitivity, the Sensitivity to Punishment and Sensitivity to Reward Questionnaire for Children,^21^ Van den Berg et al.^18^ found that higher reward responsiveness was significantly and positively associated with obesity and overeating in a cross‐sectional study of 6‐ to 13‐year‐olds. Thus, reward sensitivity subscales—like impulsivity subscales—may have implications for weight change.

### 1.2 The role of binge‐eating disorder

A minority of children meet the criteria for binge‐eating disorder (BED), which is an eating disorder characterized by frequent binge‐eating episodes (i.e., consumption of a large amount of food in a short period of time while feeling a loss of control over one's eating).^22^ BED is particularly high among children with overweight and obesity and is sometimes considered a phenotype of obesity.^22^, ^23^ Children with BED differ from those without BED in that they experience significant psychosocial and developmental impairment and have higher impulsivity towards food.^22^, ^24^, ^25^ Therefore, when studying risk factors for weight gain, it may be meaningful to examine factors separately for those with versus without BED, yet this has seldom been done, especially in children.

#### 1.2.1 Impulsivity and reward sensitivity in BED

Current literature shows a strong connection between BED and impulsivity and reward sensitivity dysfunction. A systematic review of children and adults found greater reward sensitivity in those with co‐occurring obesity and BED compared to those with obesity but without BED.^26^ Similarly, a separate study in 12‐ to 15‐year‐olds found that children with obesity that engaged in binge eating were more sensitive to reward than children with obesity that did not endorse binge eating.^27^ Another study found that women with BED were inclined to discount both delayed and probabilistic food rewards much more significantly than women with obesity but without BED and a healthy control group, suggesting higher preference for immediate rewards and reduced valuation of rewards with lower odds of acquiring them.^28^ Further, higher urgency (i.e., positive and negative urgency grouped together) and lack of perseverance have been shown to be significantly associated with binge eating in adults.^29^, ^30^ Overall, these studies show that impulsivity and reward sensitivity play a role in BED, but there is a lack of research in children, and no research on the role of impulsivity and reward sensitivity in predicting weight gain in children with versus without BED.

### 1.3 The current study

The current body of research has shown associations between impulsivity and reward sensitivity with weight and energy‐balance behaviours in children. Studies have been primarily cross‐sectional, and little research has examined the prospective effect of facets of impulsivity and reward sensitivity on weight change during late childhood or early adolescence. In addition, research has yet to examine weight change in children with BED compared to those without BED. Because there are notably more cross‐sectional studies compared to longitudinal studies, causality cannot be inferred, which precludes development of interventions and preventions for weight gain that are informed by personality research.^31^ Also, many past studies used convenience sampling rather than representative samples and were mainly focused on older adolescents and adults.

Research suggests that children with high impulsivity and reward sensitivity are more likely to be currently at a higher weight and may be more likely to engage in unhealthy energy‐balance behaviours, which could lead to weight gain over time. Therefore, as a first step, the purpose of the current study was to investigate baseline impulsivity and reward sensitivity facets as predictors of BMI‐*z* 1‐year later, controlling for baseline BMI‐*z* using data from the Adolescent Brain Cognitive Development (ABCD) Study. We used a multivariable modelling approach that examined all facets of impulsivity and reward sensitivity simultaneously. Furthermore, given the possibility of differences by BED status,^22^, ^23^, ^24^, ^25^ we ran analyses stratified by BED status (i.e., those meeting full‐ or sub‐threshold criteria for BED versus those who did not). Given this exploratory approach, we did not have specific hypotheses regarding which facets would be predictive of BMI‐*z* at 1‐year.

## 2 METHODS

### 2.1 Participants

A total of 11 875 9–10‐year‐old children and a caregiver were recruited to participate in the ABCD Study, which is primarily focused on understanding brain development across adolescence.^32^ Recruitment occurred mainly through public and private schools within 50 miles of 21 designated research sites across the United States and via referral methods, events in the community, and other schools. The 21 research sites were chosen based on having the appropriate study equipment and staff with research expertise related to the study objectives. Eligible schools were further filtered by children's demographic information (e.g., race, ethnicity, sex, percentage of students that receive lunches that are subsidized or free as an indication of socioeconomic status). Schools were randomly selected from this subset and children were recruited from this final sample of schools. Approval for this study was given by the University of California, San Diego Institutional Review Board.

### 2.2 Procedures

Children provided verbal assent while their caregiver provided written consent for participation in the study after reviewing what it entailed. Participants and their caregiver came to their nearest research site and completed the research protocol, which included a variety of components. The current study utilized data from baseline self‐report questionnaires administered to children, baseline clinical interviews administered to the caregiver, and the anthropometric assessments at baseline and 1‐year follow‐up. See Karcher and Barch^32^ for more details on the ABCD study methods.

### 2.3 Measures

#### 2.3.1 Demographics

Caregivers reported participants' age, sex, race/ethnicity, and their own annual income. Sex was categorized as male and female. Race/ethnicity was categorized as White, Black, Hispanic, Asian, or Other. Annual income was trichotomized as less than 50 000 annually, 50 000–100 000 annually, and more than 100 000 annually.

#### 2.3.2 BMI *z* ‐scores

At baseline and 1‐year follow‐up, measurements of children's height and weight were taken inperson, and BMI was calculated using the standard formula. Children's BMI (kg/m^2^) was computed by dividing weight (kg) by the square of height (m) and then converted into percentiles and *z*‐score according to the CDC 2000 Growth Chart SAS (SAS Institute, Inc, Cary, NC) software.

#### 2.3.3 Diagnostic and statistical manual of mental disorder‐5 BED diagnostic criteria

Criteria for diagnosis of eating disorders, including BED, were assessed with the Kiddie Schedule for Affective Disorders and Schizophrenia (K‐SADS) according to the diagnostic and statistical manual of mental disorder‐5 (DSM‐5) criteria.^33^ The K‐SADS was administered to the child's caregiver and assessed full‐ and sub‐threshold criteria for BED. The BED subsample represented children who met full‐ or sub‐threshold criteria for BED, given evidence of lack of meaningful difference between full‐ and sub‐threshold eating disorders.^34^ Children meeting sub‐ and full‐threshold criteria for other eating disorders were removed from analyses.

#### 2.3.4 Impulsivity

A modified version of the short form of the UPPS‐P Impulsive Behaviour Scale (UPPS‐P)^35^, ^36^ was used to assess facets of impulsivity at baseline. The UPPS‐P includes five subscales (i.e., lack of premeditation, sensation seeking, lack of perseverance, negative urgency, and positive urgency). Items were rated by children on a scale from 1 (*strongly agree*) to 4 (*strongly disagree*). Subscale scores were calculated, and higher scores represent greater impulsivity. These scales have evidenced adequate psychometric properties, as demonstrated by reliability and validity estimates in adults and children.^35^, ^37^

#### 2.3.5 Reward sensitivity

The modified Behavioural Inhibition/Behavioural Approach System (BIS/BAS Scale) was used to measure reward sensitivity at baseline.^19^, ^38^ The modified BIS/BAS includes two BAS subscales (i.e., drive and reward responsiveness) and one BIS subscale. Children rated items on a scale from 1 (*strongly agree*) to 4 (*strongly disagree*). Items were combined into the two BAS subscales and the BIS subscale, with higher scores representing greater reward sensitivity. These scales have shown good reliability and validity in adults and children.^17^, ^19^, ^38^

#### 2.3.6 Statistical analysis

This secondary data analysis of the ABCD dataset was conducted in SPSS version 28.0. The complex sampling module within SPSS was used to obtain population‐based estimates and account for the sampling design. The ABCD study provided propensity weights, which were used within complex sampling to correct for demographic selection bias. Also, the study site was used as the strata variable to account for differences by data collection site. Using the subsample function in complex samples, two multivariable general linear models were computed for children with BED and children without BED. Baseline impulsivity and reward sensitivity facets were entered simultaneously as predictors of children's BMI‐*z* scores at 1‐year follow‐up, with BMI‐*z* at baseline as a covariate. Additional demographic covariates were annual family income, sex, and race/ethnicity.

## 3 RESULTS

There were 252 children missing data on the study site who were excluded. There were 759 children who were missing BMI measures at baseline or 1‐year and removed from the data. We removed 90 children who met criteria for biologically implausible values for BMI‐*z* (BMI‐*z* <−4 or >8). There were 978 children removed for meeting full‐ or sub‐threshold criteria for another eating disorder besides BED. There were 819 children removed for missing values on independent variables or covariates. This left 8982 children for analysis.

The sample was 51.1% male and 48.9% female. There were 117 children (1.4%) who met criteria for full‐ or sub‐threshold BED. The mean BMI‐*z* score of children was 0.55 (*SE* = 0.01) at baseline and 0.61 (*SE* = 0.01) at follow‐up. The mean age was 9.50 (*SE* = 0.01). The race/ethnicity of the sample was 55.8% White, 12.2% Black, 22.0% Hispanic, 3.3% Asian, and 6.7% Other. There were 37.6% of children with annual household income less than 50 000, 31.4% between 50 000 and 100 000, and 31.0% higher than 100 000.

Results from general linear models are displayed in Table 1. BMI‐*z* at baseline was significantly associated with BMI‐*z* at 1‐year follow‐up for both subsamples. Compared to White children, Black and Hispanic children had significantly higher BMI‐*z* at 1‐year follow‐up for those without BED. Race was unrelated to BMI‐*z* in children with BED. Greater annual family income at baseline was significantly associated with BMI‐*z* at 1‐year follow‐up for both subsamples. Controlling for baseline BMI‐*z* and covariates, impulsivity and reward sensitivity were unrelated to BMI‐*z* at 1‐year follow‐up for children without BED. Yet, baseline negative urgency and positive urgency significantly predicted BMI‐*z* at 1‐year follow‐up for children with BED. Higher negative urgency and lower positive urgency predicted increases in BMI‐*z* at 1‐year follow‐up.

**TABLE 1:** Results from general linear models assessing facets of impulsivity and reward sensitivity as predictors of body mass index *z* score (BMI‐*z*) at 1‐year.

Parameter	Without BED			With BED		
	Estimate	*SE*	*p*	Estimate	*SE*	*p*
Intercept	0.13	0.05	0.007	0.60	0.29	0.04
Male vs. female	0.01	0.01	0.41	0.12	0.07	0.07
Black vs. White	0.10	0.02	<0.001	0.10	0.09	0.25
Hispanic vs. White	0.05	0.02	0.003	0.04	0.08	0.62
Asian vs. White	−0.05	0.05	0.29	‐	‐	‐
Other vs. White	0.03	0.03	0.35	0.02	0.08	0.80
Baseline BMI‐*z*	0.87	0.01	<0.001	0.69	0.08	<0.001
Annual household income	−0.02	0.01	0.02	−0.11	0.05	0.02
Negative urgency	0.002	0.003	0.55	0.04	0.02	0.02
Lack of planning	−0.003	0.003	0.28	−0.01	0.01	0.56
Sensation seeking	0.001	0.002	0.74	−0.01	0.01	0.59
Positive urgency	0.002	0.003	0.40	−0.04	0.02	0.03
Lack of perseverance	<0.001	0.003	0.99	−0.001	0.01	0.56
Reward responsiveness	−0.003	0.003	0.85	0.01	0.02	0.42
Drive	0.003	0.003	0.32	0.001	0.01	0.96
Inhibition	<0.001	0.002	0.28	0.003	0.01	0.82

Abbreviations: BED, binge‐eating disorder; BMI‐*z*, body mass index *z*‐score.

## 4 DISCUSSION

This secondary data analysis leveraged the longitudinal ABCD study to explore facets of impulsivity and reward sensitivity as predictors of BMI‐*z* scores 1‐year later in pre‐adolescents, stratified by BED status. Neither impulsivity nor reward sensitivity predicted weight change in children without BED. Yet, among children with BED, baseline negative urgency and positive urgency prospectively predicted increased BMI‐*z* after 1 year, controlling for BMI‐*z* at baseline. No other facets of impulsivity or reward sensitivity significantly predicted BMI‐*z* at 1‐year follow‐up among children with BED.

The finding of the importance of negative urgency in BMI‐*z* change in children with BED aligns with previous work. For example, studies have found that negative urgency was significantly associated with elevated binge eating severity^28^, ^29^, ^30^ and increases in hedonic hunger.^39^ This is also consistent with the affect regulation model of binge eating, which shows that binge eating serves to reduce negative affect experiences.^40^, ^41^ It is possible that negative urgency is associated with greater severity of BED, including greater binge‐eating frequency, uncontrolled eating, and less likelihood of full recovery from binge‐eating symptoms, which may lead to increased weight gain over time.

Oppositely, greater positive urgency was associated with less weight gain among children with BED. This finding is somewhat inconsistent to other research, which found a positive association between positive urgency and weight gain.^42^ However, this was in a sample of young adults that presumably do not have BED. As such, children with BED (who often have overweight/obesity and psychiatric comorbidity) appear to have a different relationship between positive urgency and weight gain. One possible reason for this could be that greater positive urgency increases the likelihood of engagement in vigorous physical activity, which was found in previous research of adolescents.^13^

Findings for null results in children without BED in the current study suggest that other factors (e.g., family, environment) may be more important during this timeframe in predicting weight gain. Indeed, we found that race/ethnicity and income were salient risk factors for weight gain in children without BED. Further, it is possible the relationship between impulsivity, reward sensitivity, and weight‐related behaviours is more complex, especially for children without BED. In addition, impulsivity and reward sensitivity may become more important predictors of weight gain for individuals without BED during late adolescence and early adulthood as the brain and behaviours develop and change. More research will be necessary to study behavioural mediators of the prospective associations between impulsivity and reward sensitivity and BMI‐*z* in pre‐adolescents, including dietary intake, eating patterns, and physical activity. It will also be important to study predictive differences among other psychiatric and demographic subgroups in future research.

### 4.1 Strengths and limitations

The primary strengths of this study were the longitudinal design, a structured interview assessment of BED diagnostic criteria, and an objective measurement of height and weight among a representative sample of children in the United States. Yet, the study had some limitations. First, the questionnaires for impulsivity and reward sensitivity were self‐reported, which may have been subject to self‐report biases. Second, use of BMI has inherent limitations and does not allow us to distinguish between muscle mass weight and fat mass weight.^43^ Other measures including body fat percentage, waist circumference, and muscle mass should be considered in future research. Third, results of diagnostic interviews that assessed BED may be somewhat biased since they were only given to caregivers. Future studies should use a multi‐rater approach to assess BED criteria with both child and caregiver.

## 5 CONCLUSIONS AND FUTURE DIRECTIONS

Our findings support that, of all the facets that were assessed, negative urgency and positive urgency were salient predictors of weight gain in children with BED. This is important in developing theoretical models and interventions for weight gain prevention in children with BED and children high in negative urgency and low in positive urgency. In addition, these personality traits should be studied further to better understand their aetiology in children for screening purposes and address their implications to reduce paediatric obesity, particularly in children with BED. Clinicians should assess for negative urgency, positive urgency, and for BED to determine at‐risk children for weight gain. Specifically, novel interventions focused on emotion regulation when feeling impulsive might be useful for children with BED to prevent weight gain.

## FUNDING INFORMATION

This project was supported in part by grant K01DK124435 and K23DK128568, both from the National Institute of Diabetes and Digestive and Kidney Diseases Award Number (NIDDK).

## CONFLICT OF INTEREST

The authors have no conflicts of interest to disclose.

## References

[1] WHO . *Obesity and overweight*. World Health Organization. Accessed July 7, 2021. https://www.who.int/news-room/fact-sheets/detail/obesity-and-overweight.

[2] Crowley DI , Khoury PR , Urbina EM , Ippisch HM , Kimball TR . Cardiovascular impact of the pediatric obesity epidemic: higher left ventricular mass is related to higher body mass index. J Pediatr. 2011;158(5):709‐714.e1. doi:10.1016/j.jpeds.2010.10.016 21147488

[3] CDC . *Childhood obesity causes & consequences*. Centers for Disease Control and Prevention. Accessed July 7, 2021. https://www.cdc.gov/obesity/childhood/causes.html.

[4] Dreyfuss M , Caudle K , Drysdale AT , et al. Teens impulsively react rather than retreat from threat. Dev Neurosci. 2014;36(3–4):220‐227. doi:10.1159/000357755 24821576 PMC4125471

[5] Thamotharan S , Lange K , Zale EL , Huffhines L , Fields S . The role of impulsivity in pediatric obesity and weight status: a meta‐analytic review. Clin Psychol Rev. 2013;33:253‐262. doi:10.1016/j.cpr.2012.12.001 23313762

[6] Davis C , Patte K , Levitan R , Reid C , Tweed S , Curtis C . From motivation to behaviour: a model of reward sensitivity, overeating, and food preferences in the risk profile for obesity. Appetite. 2007;48(1):12‐19. doi:10.1016/j.appet.2006.05.016 16875757

[7] Kaiser A , Holz NE , Banaschewski T , et al. A developmental perspective on facets of impulsivity and brain activity correlates from adolescence to adulthood. Biol Psychiatry Cogn Neurosci Neuroimaging. 2022. 10.1016/j.bpsc.2022.02.003 PMC9636026 35182817

[8] Harden KP , Mann FD , Grotzinger AD , et al. Developmental differences in reward sensitivity and sensation seeking in adolescence: testing sex‐specific associations with gonadal hormones and pubertal development. J Personal Soc Psychol. 2018;115(1):161. doi:10.1037/pspp0000172 PMC5932293 29094961

[9] Kim S , Lee D . Prefrontal cortex and impulsive decision making. Biol Psychiatry. 2011;69:1140‐1146. doi:10.1016/j.biopsych.2010.07.005 20728878 PMC2991430

[10] Bakhshani NM . Impulsivity: a predisposition toward risky behaviors. Int J High Risk Behav Addict. 2014;3(2):e20428. doi:10.5812/ijhrba.20428 25032165 PMC4080475

[11] Cyders MA , Smith GT . Mood‐based rash action and its components: positive and negative urgency. Personal Individ Differ. 2007;43:839‐850. doi:10.1016/j.paid.2007.02.008

[12] Whiteside SP , Lynam DR . The five factor model and impulsivity: using a structural model of personality to understand impulsivity. Personal Individ Differ. 2001;30:669‐689. doi:10.1016/S0191-8869(00)00064-7

[13] Smith KE , Lavender JM , Leventhal AM , Mason TB . Facets of impulsivity in relation to diet quality and physical activity in adolescence. Int J Environ Res Public Health. 2021;18(613):1‐10. doi:10.3390/ijerph18020613 PMC7828222 33445815

[14] Goodnight J . Reward sensitivity. In: Bornstein M , ed. The Sage Encyclopedia of Lifespan Human Development. Sage; 2018:1854‐1855. doi:10.4135/9781506307633.n690

[15] De Decker A , Sioen I , Verbeken S , Braet C , Michels N , De Henauw S . Associations of reward sensitivity with food consumption, activity pattern, and BMI in children. Appetite. 2016;100:189‐196. doi:10.1016/j.appet.2016.02.028 26898320

[16] Jonker NC , Van Malderen E , Glashouwer KA , et al. No differential reward responsivity and drive, punishment sensitivity or attention for cues signaling reward or punishment in adolescents with obesity. Front Psychol. 2019;10(2363):1‐16. doi:10.3389/fpsyg.2019.02363 31695649 PMC6817582

[17] Mason TB , Do B , Dunton G . Interactions of approach motivation and self‐regulation in relation to obesity in children. Eat Weight Disord. 2021;26:85‐92. doi:10.1007/s40519-019-00817-2 31784947 PMC7255926

[18] Van den Berg L , Pieterse K , Malik JA , et al. Association between impulsivity, reward responsiveness and body mass index in children. Int J Obes. 2011;35:1301‐1307. doi:10.1038/ijo.2011.116 21694699

[19] Carver CS , White TL . Behavioral inhibition, behavioral activation, and affective responses to impending reward and punishment: the BIS/BAS scales. J Personal Soc Psychol. 1994;67(2):319‐333. doi:10.1037/0022-3514.67.2.319

[20] De Cock N , Van Lippevelde W , Vervoort L , et al. Sensitivity to reward is associated with snack and sugar‐sweetened beverage consumption in adolescents. Eur J Nutr. 2016;55(4):1623‐1632. doi:10.1007/s00394-015-0981-3 26163856

[21] Colder CR , O'Connor RM . Gray's reinforcement sensitivity model and psychopathology: laboratory and questionnaire assessment of the BAS and BIS. J Abnorm Child Psychol. 2004;32(4):435‐451. doi:10.1023/B:JACP.0000030296.54122.b6 15305548

[22] American Psychiatric Association . Diagnostic and Statistical Manual of Mental Disorders. 5th ed. American Psychiatric Association; 2013. doi:10.1176/appi.books.9780890425596

[23] Dalton M , Blundell J , Finlayson G . Effect of BMI and binge eating on food reward and energy intake: further evidence for a binge eating subtype of obesity. Obes Facts. 2013;6:348‐359. doi:10.1159/000354599 23970144 PMC5644679

[24] Bohon C . Binge eating disorder in children and adolescents. Child Adolesc Psychiatr Clin N Am. 2019;28(4):549‐555. doi:10.1016/j.chc.2019.05.003 31443873 PMC6709690

[25] Marzilli E , Cerniglia L , Cimino S . A narrative review of binge eating disorder in adolescence: prevalence, impact, and psychological treatment strategies. Adolesc Health Med Ther. 2018;9:17‐30. doi:10.2147/AHMT.S148050 29379325 PMC5759856

[26] Schag K , Schönleber J , Teufel M , Zipfel S , Giel KE . Food‐related impulsivity in obesity and binge eating disorder—a systematic review. Obes Rev. 2013;14(6):477‐495. doi:10.1111/obr.12017 23331770

[27] Nederkoorn C , Braet C , Van Ejis Y , Tanghe A , Jansen A . Why obese children cannot resist food: the role of impulsivity. Eat Behav. 2006;7:315‐322. doi:10.1016/j.eatbeh.2005.11.005 17056407

[28] Manwaring JL , Green L , Myerson J , Strube MJ , Wilfley DE . Discounting of various types of rewards by women with and without binge eating disorder: evidence for general rather than specific differences. Psychol Rec. 2011;61(4):561‐582. doi:10.1007/BF03395777 24039301 PMC3770473

[29] Kenny TE , Singleton C , Carter JC . An examination of emotion‐related facets of impulsivity in binge eating disorder. Eat Behav. 2019;32:74‐77. doi:10.1016/j.eatbeh.2018.12.006 30654194

[30] Thomsen KR , Callesen MB , Hesse M , et al. Impulsivity traits and addiction‐related behaviors in youth. J Behav Addict. 2018;7(2):317‐330. doi:10.1556/2006.7.2018.22 29642723 PMC6174598

[31] Gerlach G , Herpertz S , Loeber S . Personality traits and obesity: a systematic review. Obes Rev. 2015;16(1):32‐63. doi:10.1111/obr.12235 25470329

[32] Karcher NR , Barch DM . The ABCD study: understanding the development of risk for mental and physical health outcomes. Neuropsychopharmacology. 2021;46:131‐142. doi:10.1038/s41386-020-0736-6 32541809 PMC7304245

[33] Ambrosini PJ . Historical development and present status of the schedule for affective disorders and schizophrenia for school‐age children (K‐SADS). J Am Acad Child Adolesc Psychiatry. 2000;39(1):49‐58. doi:10.1097/00004583-200001000-00016 10638067

[34] Forbush KT , Chen PY , Hagan KE , et al. A new approach to eating‐disorder classification: using empirical methods to delineate diagnostic dimensions and inform care. Int J Eat Disord. 2018;51(7):710‐721. doi:10.1002/eat.22891 30132954

[35] Cyders MA , Littlefield AK , Coffey S , Karyadi KA . Examination of a short English version of the UPPS‐P impulsive behavior scale. Addict Behav. 2014;39:1372‐1376. doi:10.1016/j.addbeh.2014.02.013 24636739 PMC4055534

[36] Barch DM , Albaugh MD , Avenevoli S , et al. Demographic, physical and mental health assessments in the adolescent brain and cognitive development study: rationale and description. Dev Cogn Neurosci. 2018;32:55‐66. doi:10.1016/j.dcn.2017.10.010 29113758 PMC5934320

[37] Geurten M , Catale C , Gay P , Deplus S , Billieux J . Measuring impulsivity in children: adaptation and validation of a short version of the UPPS‐P impulsive behaviors scale in children and investigation of its links with ADHD. J Atten Disord. 2021;25(1):105‐114. doi:10.1177/1087054718775831 29771172

[38] Pagliaccio D , Luking KR , Anokhin AP , et al. Revising the BIS/BAS scale to study development: measurement invariance and normative effects of age and sex from childhood through adulthood. Psychol Assess. 2016;28(4):429‐442. 26302106 10.1037/pas0000186 PMC4766059

[39] Mason TB , Dunton GF , Gearhardt AN , Leventhal AM . Emotional disorder symptoms, anhedonia, and negative urgency as predictors of hedonic hunger in adolescents. Eat Behav. 2020;36:101343. doi:10.1016/j.eatbeh.2019.101343 31715461 PMC7044051

[40] Berg KC , Cao L , Crosby RD , et al. Negative affect and binge eating: reconciling differences between two analytic approaches in ecological momentary assessment research. Int J Eat Disord. 2017;50(10):1222‐1230. doi:10.1002/eat.22770 28851137 PMC8686165

[41] Schaefer LM , Smith KE , Anderson LM , et al. The role of affect in the maintenance of binge‐eating disorder: evidence from an ecological momentary assessment study. J Abnorm Psychol. 2020;129(4):387‐396. doi:10.1037/abn0000517 32212743 PMC7174093

[42] Bjorlie K , Fazzino TL . Impulsivity as a risk factor for weight gain and body roundness change among college freshmen. Eat Behav. 2020;39:1‐7. doi:10.1016/j.eatbeh.2020.101435 PMC8796869 33022473

[43] Nuttall FQ . Body mass index: obesity, BMI, and health: a critical review. Nutr Today. 2015;50(3):117‐128. doi:10.1097/NT.0000000000000092 27340299 PMC4890841

